# Supplementary material for: Content-rich biological network constructed by mining PubMed abstracts
Source: BMC Bioinformatics. 2004 Oct 8;5:147. doi: 10.1186/1471-2105-5-147 (PMC528731; doi:10.1186/1471-2105-5-147)
Supplement: Additional File 2 — The original results of the above study (non-essential files are deleted to keep the file size under the limit set by BMC bioinformatics). [file 1471-2105-5-147-S2.bz2 › chilibotAdditionalFile2/dip05/637ID10917520E11024/html/FCER1A_IGHE.html]

 


 **FCER1A** and **IGHE** 
  
Found 14 abstracts in PubMed, retrieved 05.  
 

 What does Google say? 
 PDF only 
| .edu only 

---

**Interactive relationship** (e.g. stimulation, inhibition, etc)

**Inhibitory relationship**- This antibody recognises specifically free but not FcERI  [ **FCER1A** ]  bound IgE  [ **IGHE** ]  thus preventing ligand binding and subsequent cell activation.  Ref: 12836193 Med Sci Paris , 2003
**Neutral relationship**- The recent elucidation of the tridimensional structure of the complex between IgE  [ **IGHE** ]  and FcERI  [ **FCER1A** ]  provides unexpected information regarding the mechanism of assembly of the complex, which now can be used to design small chemical compounds capable of specifically inhibiting this interaction.  Ref: 12836193 Med Sci Paris , 2003
- Interaction of an allergen with its specific IgE  [ **IGHE** ]  bound to FcERI  [ **FCER1A** ]  triggers cell activation, which induces the release of numerous mediators that are responsible for allergic manifestations.  Ref: 12836193 Med Sci Paris , 2003
- This four YAC contig includes other genes with immune functions including the  **FCER1A**  gene, which encodes the alpha subunit of the IgE  [ **IGHE** ]  high affinity Fc receptor and the IFI 16 gene, an interferon gamma induced gene.  Ref: 8613143 Immunogenetics, 1996

**Non-interactive relationship** (e.g. studied together, co-existance, homology, etc.)

- Aggregation of the high affinity receptor for IgE  [ **IGHE** ]  FceRI  [ **FCER1A** ]  on mast cells results in the rapid phosphorylation of tyrosines on the beta and gamma chains of the receptor by the Src family kinase Lyn, which initiates the signaling cascades leading to secretion of inflammatory mediators.  Ref: 11388899 J Biochem Tokyo , 2001
- We synthesized various analogues of indolyl, naphthyl and phenylethyl substituted halopyridyl, thiazolyl and benzothiazolyl thioureas and examined their in vitro effects on the high affinity IgE  [ **IGHE** ]  receptor FcERI  [ **FCER1A** ]  mediated mast cell leukotriene release.  Ref: 12565956 Bioorg Med Chem Lett, 2003
- This ELISA used FcERI  [ **FCER1A** ]  IgG for capture and peroxidase labelled goat polyclonal antibody to human IgE  [ **IGHE** ]  for detection.  Ref: 8760275 Mol Immunol< MedlineTA>Mol Immunol, 2003
- The high affinity receptor for IgE  [ **IGHE** ] , FcERI  [ **FCER1A** ] , is at the core of the allergic reaction.  Ref: 12836193 Med Sci Paris , 2003
